# Etoposide Induces ATM-Dependent Mitochondrial Biogenesis through AMPK Activation

**DOI:** 10.1371/journal.pone.0002009

**Published:** 2008-04-23

**Authors:** Xuan Fu, Shan Wan, Yi Lisa Lyu, Leroy F. Liu, Haiyan Qi

**Affiliations:** Department of Pharmacology, UMDNJ-Robert Wood Johnson Medical School, Piscataway, New Jersey, United States of America; National Institute on Aging, United States of America

## Abstract

**Background:**

DNA damage such as double-stranded DNA breaks (DSBs) has been reported to stimulate mitochondrial biogenesis. However, the underlying mechanism is poorly understood. The major player in response to DSBs is ATM (ataxia telangiectasia mutated). Upon sensing DSBs, ATM is activated through autophosphorylation and phosphorylates a number of substrates for DNA repair, cell cycle regulation and apoptosis. ATM has been reported to phosphorylate the α subunit of AMP-activated protein kinase (AMPK), which senses AMP/ATP ratio in cells, and can be activated by upstream kinases. Here we provide evidence for a novel role of ATM in mitochondrial biogenesis through AMPK activation in response to etoposide-induced DNA damage.

**Methodology/Principal Findings:**

Three pairs of human ATM+ and ATM- cells were employed. Cells treated with etoposide exhibited an ATM-dependent increase in mitochondrial mass as measured by 10-N-Nonyl-Acridine Orange and MitoTracker Green FM staining, as well as an increase in mitochondrial DNA content. In addition, the expression of several known mitochondrial biogenesis regulators such as the major mitochondrial transcription factor NRF-1, PGC-1α and TFAM was also elevated in response to etoposide treatment as monitored by RT-PCR. Three pieces of evidence suggest that etoposide-induced mitochondrial biogenesis is due to ATM-dependent activation of AMPK. First, etoposide induced ATM-dependent phosphorylation of AMPK α subunit at Thr172, indicative of AMPK activation. Second, inhibition of AMPK blocked etoposide-induced mitochondrial biogenesis. Third, activation of AMPK by AICAR (an AMP analogue) stimulated mitochondrial biogenesis in an ATM-dependent manner, suggesting that ATM may be an upstream kinase of AMPK in the mitochondrial biogenesis pathway.

**Conclusions/Significance:**

These results suggest that activation of ATM by etoposide can lead to mitochondrial biogenesis through AMPK activation. We propose that ATM-dependent mitochondrial biogenesis may play a role in DNA damage response and ROS regulation, and that defect in ATM-dependent mitochondrial biogenesis could contribute to the manifestations of A-T disease.

## Introduction

Mitochondria play important roles in ATP synthesis and apoptosis [1]–[4]. A number of human diseases are linked to mutations of the mitochondrial genome [1]–[4]. Among these are premature ageing, cancer, diabetes mellitus, and a variety of syndromes involving the muscles and the central nervous system [1]–[4].

Human cells contain a few hundred to thousand mitochondria per cell, and each mitochondrion has 1–10 copies of the 16 kb double-stranded circular DNA that encodes 37 genes. Mitochondrial biogenesis involves mitochondrial DNA (mtDNA) replication and mitochondrial mass increase. Due to limited coding capacity of mtDNA, mitochondria rely largely on nuclear genes (over 1000 genes) for their proliferation [5]. Mitochondrial biogenesis therefore requires complex coordination between the nuclear and mitochondrial genomes. This is largely achieved through the peroxisome proliferation activator receptor gamma-coactivator 1α (PGC-1α) [6]. PGC-1α upregulates two nuclear transcription factors known as NRF-1 and -2 (nuclear respiratory factors 1 and 2) which activate transcription of nuclear-encoded mitochondrial genes [6]–[8]. PGC-1α also upregulates mitochondrial transcription factor A, TFAM, which stimulates transcription of mitochondrial genes [6], [8]–[10].

Mitochondrial biogenesis has been reported to be regulated by the energy state of cells through AMP-activated protein kinase (AMPK) [11], [12]. Upon energy depletion, activated AMPK turns off ATP-consuming processes such as synthesis of lipids, carbohydrates, and proteins, and turns on ATP-generating pathways including mitochondrial biogenesis [12], [13]. AMPK is allosterically stimulated by AMP and is then activated through phosphorylation at Thr172 of the AMPK catalytic α subunit by upstream kinase(s) such as tumor suppressor LKB1 and CaMKK (Ca2+/calmodulin-dependent protein kinase kinase) [14]–[18]. AMPK is exquisitely sensitive to AMP/ATP ratio [19] . AMPK directly activates PGC-1α by phosphorylation at Thr177 and Ser538 [20], and is also known to upregulate DNA binding activity of NRF-1 [13].

Mitochondrial biogenesis has been reported to increase in response to DNA damage [21], [22]. For example, DNA topoisomerase II-targeting anticancer drugs (e.g. doxorubicin, mitoxantrone and etoposide), which are known to induce DNA double-strand breaks (DSBs), have been reported to upregulate the abundance of mitochondria. However, the underlying mechanism is not understood [21], [22]. The major player in response to DSBs is ATM (ataxia telangiectasia mutated), which belongs to the PIKK (phosphoinositide 3-kinase related kinase) family of protein kinases [23], [24]. ATM has been shown to phosphorylate AMPK α subunit *in vitro* [25]. In addition, phosphorylation of the AMPK α subunit stimulated by AICAR, an AMPK activator that can be converted to ZMP in cells to mimic AMP, has been shown to be ATM-dependent [26]. These results point to the possibility that ATM may regulate mitochondrial biogenesis through AMPK.

In the current study, we demonstrate that etoposide upregulates mitochondrial biogenesis in an ATM-dependent manner. In addition, ATM-dependent mitochondrial biogenesis is at least in part mediated through AMPK activation.

## Results

**Figure 1:**
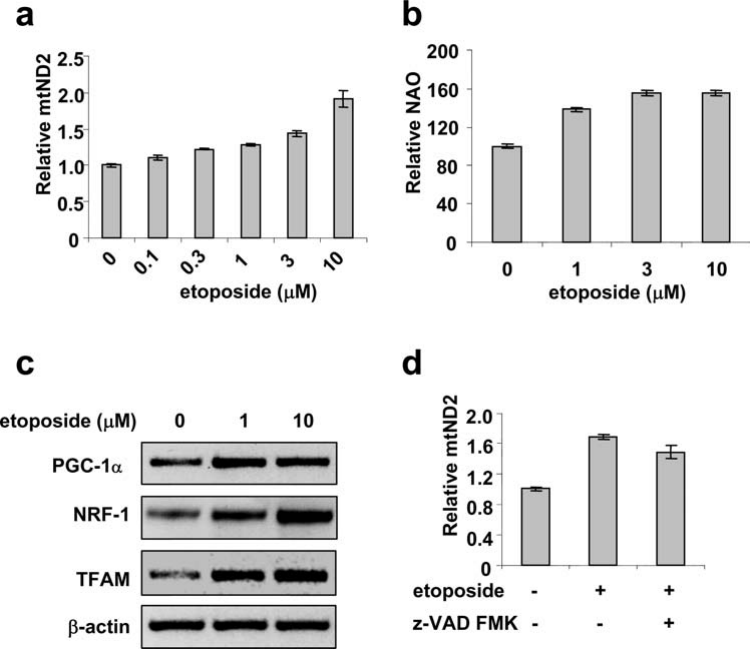
Etoposide induces mitochondrial biogenesis. HeLa cells were treated with various concentrations of etoposide for 16 hrs. mtDNA content (a) and mitochondrial mass (b) were determined as described in the Materials and Methods. Data are presented as mean±SEM (n = 3). c. The mRNA levels of PGC1α, NRF−1 and TFAM were determined by RT-PCR. β-actin was used as a control for RT-PCR. d. HeLa cells were treated with 10 µM etoposide in the presence or absence of the caspase inhibitor z-VAD-FMK (20 µM) for 16 hrs and mtDNA content was then determined.

In order to confirm that DNA damage induces mitochondrial biogenesis, we employed etoposide, a topoisomerase II poison known to induce DSBs and activate ATM [27], [28]. HeLa cells were treated with etoposide for 16 hrs, mitochondrial DNA content and mitochondrial mass were then determined. As shown in Fig. 1a, etoposide induced a dose-dependent increase in the copy number of the mitochondrial NADH dehydrogenase subunit 2 (mtND2) gene (normalized to that of the nuclear 18S rDNA) in HeLa cells as measured by quantitative real time PCR. The increase in mtND2 gene copy number in HeLa cells paralleled an increase in the mitochondrial mass as evidenced by increased fluorescence staining of cardiolipin in the inner mitochondrial membrane using 10-N-Nonyl-Acridine Orange (NAO) (Fig. 1b). These results suggest that both mitochondrial DNA content and mass were increased in response to etoposide treatment. To provide more evidence that mitochondrial biogenesis was activated by etoposide, the steady-state mRNA levels of several known regulators of mitochondrial biogenesis were determined. As shown in Fig. 1c, the mRNA level of PGC-1α, the master regulator for mitochondrial biogenesis, increased two-fold in etoposide-treated HeLa cells compared to that in control cells (DMSO-treated) as measured by RT-PCR. The mRNA level of NRF-1, a transcription regulator for nuclear-encoded mitochondrial genes [7], was also upregulated by etoposide. TFAM, a regulator of mitochondrial DNA replication and transcription, has been shown to be controlled by both NRF-1 and PGC-1α [6], [29]. As shown in Fig. 1c, the mRNA level of TFAM was induced to a similar extent as that of PGC-1α. Taken together, these results suggest that etoposide stimulates key mitochondrial transcription regulators and induces mitochondrial biogenesis.

High concentrations of etoposide are known to induce apoptosis [30]. To test whether the apoptotic pathway is involved, we blocked caspase activation using a pan-caspase inhibitor z-VAD-FMK and found that the increase in mtDNA content in etoposide-treated cells was not significantly affected (Fig. 1d). These results suggest that mitochondrial biogenesis induced by etoposide is not dependent on caspase activation.

**Figure 2:**
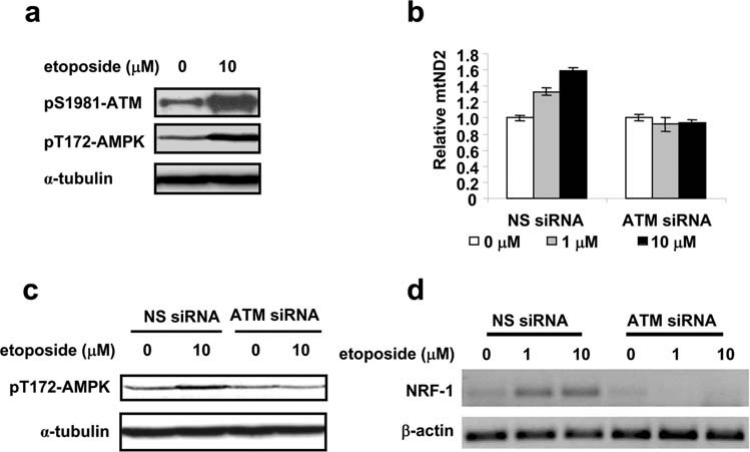
Etoposide-induced mitochondrial biogenesis is ATM-dependent. a. Activation of ATM and AMPK were determined by Western blotting using specific antibodies against phosphorylated ATM at Ser1981 and phosphorylated AMPK at Thr172. α-tubulin was used as loading controls. HeLa cells were treated with 10 µM etoposide for 16 hrs. b–c. HeLa cells transfected with ATM siRNA or non-specific siRNA (NS siRNA) were treated with etoposide for 16 hrs. The mtDNA content was determined by quantitative real-time PCR (b). Phosphorylated AMPK at Thr172 was monitored by Western blotting (c). d. ATM siRNA and NS siRNA HeLa cells were treated with etoposide for 16 hrs. RNA was then isolated and the NRF-1 mRNA level was determined using RT-PCR.

Mitochondrial biogenesis has been reported to be regulated by AMPK [11]–[13], [20]. To test whether AMPK is involved in etoposide-induced mitochondrial biogenesis, the activation of AMPK was monitored in etoposide-treated HeLa cells by immunoblotting using anti-phospho-Thr172 AMPK antibody. As shown in Fig. 2a, etoposide treatment activated ATM as evidenced by ATM autophosphorylation at Ser1981, and AMPK as evidenced by an increase in phosphorylation of AMPK α at Thr172.

It is well known that DSBs induced by etoposide activate ATM kinase activity [27], [28]. It has also been reported that in response to IGF-1 stimulation, purified ATM phosphorylates and activates AMPK [25]. We therefore hypothesize that etoposide-induced mitochondrial biogenesis is mediated through the ATM activation. To test this hypothesis, activation of AMPK and mitochondrial DNA content were monitored in two pairs of ATM+/ATM− cell lines: ATM siRNA knockdown HeLa cells and non-specific siRNA-transfected control HeLa cells, as well as ATM cDNA-complemented human A-T fibroblast YZ5 (ATM+) and its vector plasmid-transfected control pEBS7 (ATM−). As shown in Fig. 2b and 2c, as well as supplementary Figures S1 and S2, etoposide induced an increase in AMPK α phosphorylation at Thr172 and in mitochondrial DNA content in ATM+ but not in ATM− cells. Furthermore, NRF-1 mRNA induction stimulated by etoposide was also abolished in ATM siRNA knockdown HeLa cells as shown in Fig. 2d. These results suggest that AMPK activation, NRF-1 upregulation and subsequent mitochondrial biogenesis in response to etoposide treatment are dependent on ATM.

**Figure 3:**
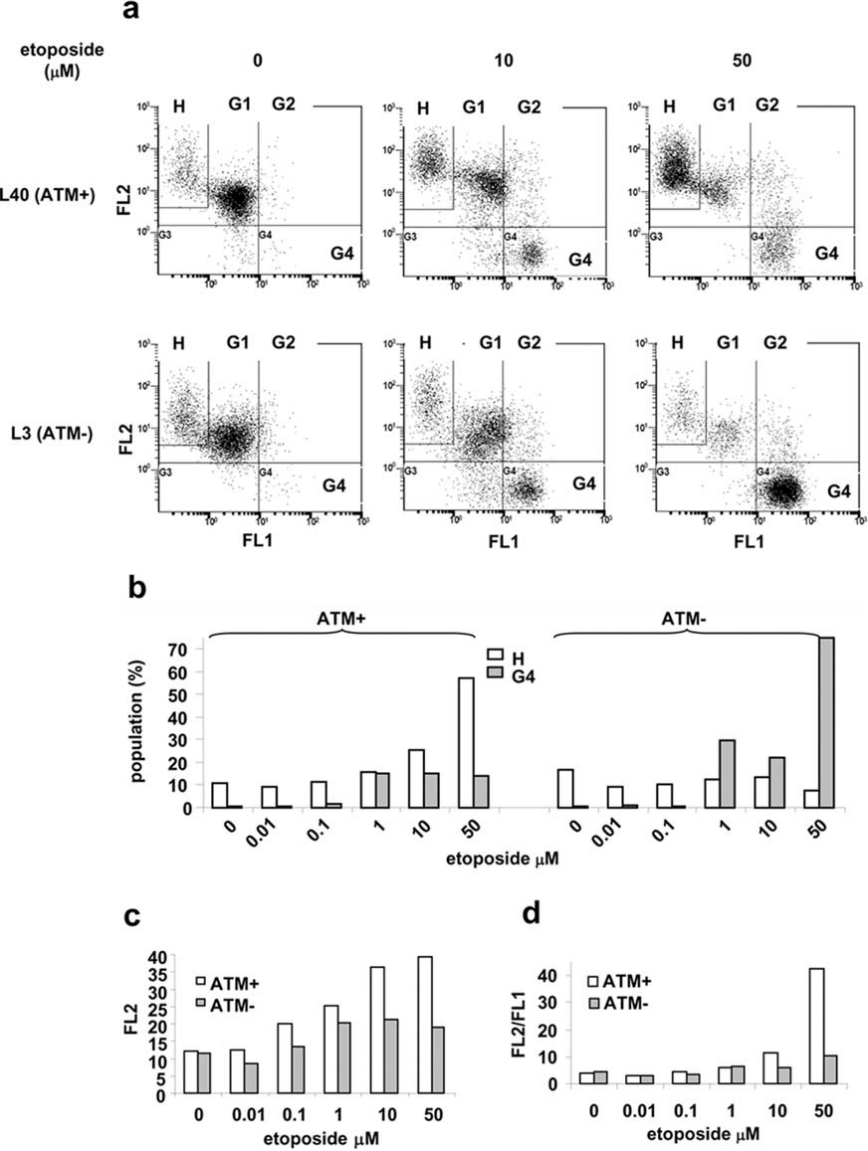
ATM increases mitochondrial membrane potential in surviving population. a. L40 (ATM+) and L3 (ATM−) cells were treated with indicated concentrations of etoposide for two days, followed by JC-1 staining and FACS analysis. Data were collected from normal living cell population that was gated according to the controls (no treatment) based on forward and side scatterings. b–d. Results were obtained from supplementary Fig. S4. b. Percentages of the H and the G4 populations in ATM+ and ATM− cells were plotted against etoposide concentrations. c. The mean intensities of FL2 in G1 population were plotted against etoposide concentrations. d. The ratios of the mean intensity of FL2 to the mean intensity of FL1 for G1 cells (including H cells) were plotted against etoposide. Data are a representative of three independent experiments.

Mitochondrial biogenesis could amplify and repopulate functional mitochondria, which is expected to increase both the mitochondrial mass and the overall mitochondrial membrane potential [31], [32]. To test this idea, we measured mitochondrial mass and membrane potential. The pair of L40 (ATM+) and L3 (ATM−) lymphoblastoid cells were employed. As shown in supplementary Figure S3, L40 (ATM+) cells exhibited increased mitochondrial mass as compared to L3 (ATM−) cells in response to etoposide treatment. To measure mitochondrial membrane potential, cells were treated with etoposide and stained with JC-1 which accumulates as aggregates in mitochondria of high membrane potential (red, detected in FL2) and exists as monomer in cytoplasm (green, detected in FL1) [33]. As shown in Fig. 3a, 3b and supplementary Figure S4, with increasing etoposide concentrations (0, 10 and 50 µM), a cell population with high FL2 intensity (H) was increased in ATM+ cells (10.8%, 25.6% and 57.2%, respectively), while decreased in ATM- cells (16.9%, 13.3% and 7.3%, respectively). The mean intensity of FL2 in G1 population (including H), which represents normal live cells (as shown in Fig. 3a), also increased in ATM+ cells as shown in Fig 3c. The FL2/FL1 values (indicative of mitochondrial membrane potential) in G1 population were thus increased in response to etoposide treatment in an ATM-dependent manner (Fig. 3d). It appears that etoposide induced ATM-dependent mitochondrial biogenesis leads to an overall increase in mitochondria of good quality in surviving cells. Our results provide a possible explanation for recent observations that A-T lymphoblastoid cells from patients exhibit lower mitochondrial mass staining and lower membrane potential [34] and that cerebellum, lung, marrow and muscle from A-T mice exhibit lower mtDNA copy number [35].

It was also noted that the G4 cell population with depolarized mitochondria was increased in etoposide-treated ATM+ cells in a dose-dependent manner (0.43%, 14.1% and to 14.9% in response to 0, 10 and 50 µM etoposide, respectively) as shown in Fig. 3, and supplementary Figure S4. However, the increase in the G4 population is even more significant in ATM- cells (0.74%, 22.8% and 74.9% in response to 0, 10 and 50 µM etoposide, respectively) comparing to that in ATM+ cells. MTT assay also showed that ATM+ cells were more resistant to etoposide than ATM- cells (Supplementary Figure S5). These results suggest that in the absence of ATM, DSBs induced by etoposide triggers a cell death pathway involving depolarization of mitochondria. ATM appears to protect cells from this cell death pathway through its role in mitochondrial biogenesis and/or DNA repair.

**Figure 4:**
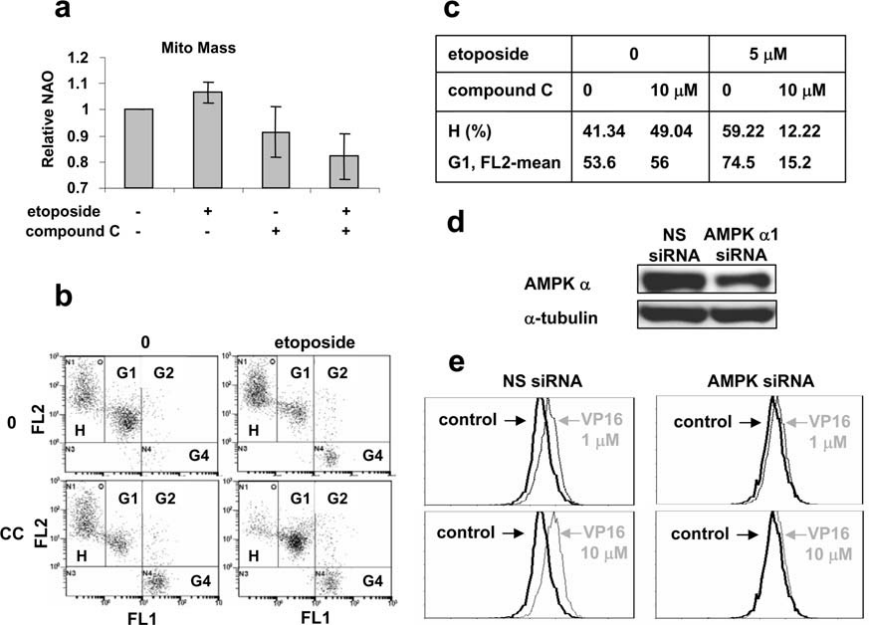
Inhibition of AMPK abolishes etoposide-induced mitochondrial biogenesis. a. Compound C blocks etoposide-induced increase in mitochondrial mass. L40 (ATM+) cells were treated with DMSO (control), 5 µM etoposide, 10 µM compound C, and 5 µM etoposide plus 10 µM compound C for 18 hrs. Cells were then stained by 1 µM NAO for FACS analysis. Data were obtained from three independent experiments. b–c. Compound C inhibits the increase in FL2 intensity of survival cells, as well as the increase of the H cell population. L40 (ATM+) cells were treated as described in a. Cells were then stained with JC-1 prior to FACS analysis. d. HeLa cells were transfected with siRNA against AMPK α or non-specific siRNA (NS). Knockdown of AMPK α was monitored by Western blotting using an antibody against AMPK α. e. AMPK α siRNA inhibits the increase in mitochondrial mass induced by etoposide. Cells were treated with indicated concentrations of etoposide (VP16) for 18 hrs. Cells were fixed by 60% ethanol and stained with MitoTracker Green FM prior to FACS analysis. 10,000 cells were analyzed.

To test whether AMPK plays a critical role in ATM-dependent mitochondrial biogenesis, the AMPK specific inhibitor, compound C, was employed [36]. Compound C was identified through a high-throughput *in vitro* assay from a library containing more than 10, 000 small molecules [36]. It is a potent reversible AMPK inhibitor that competes with ATP with *K*_i_ = 109±16 nM in the absence of AMP [36]. As shown in Fig. 4a, compound C (10 µM) blocked the increase in mitochondrial mass induced by etoposide as measured by NAO staining of living cells. Furthermore, the increase in H population and in the mean FL2 intensity of G1 population induced by etoposide (5 µM) were both blocked by compound C (Fig. 4b and 4c). In order to confirm these results, knockdown of AMPK using siRNA specifically against the AMPK α subunit in HeLa cells was carried out. As shown in Fig. 4d and 4e, AMPK α was knocked down by the siRNA, which abolished mitochondrial mass increase induced by etoposide at 1 and 10 µM. These results suggest that ATM regulates mitochondrial biogenesis through AMPK, which is also responsible for the increase in mitochondrial membrane potential of the G1 population.

**Figure 5:**
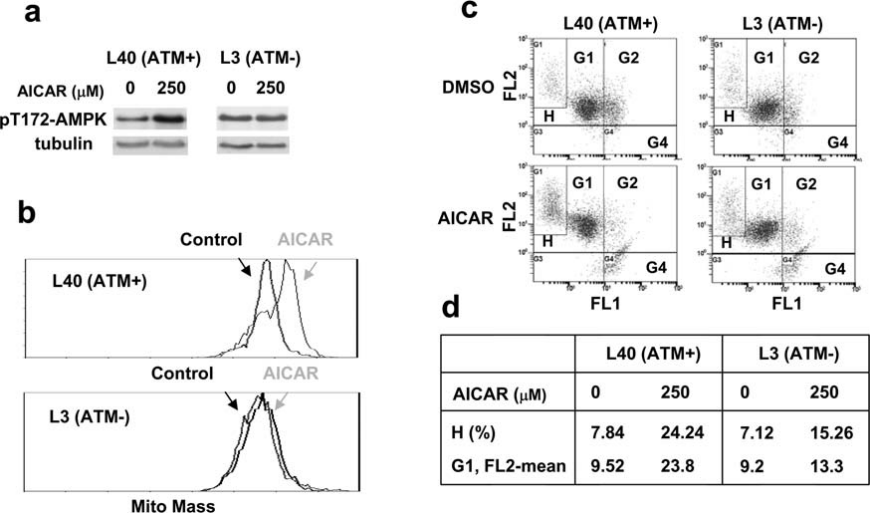
AICAR induces ATM-dependent mitochondrial biogenesis. a. Cells were treated with 250 µM AICAR for 100 min. Their lysates were then analyzed for activation of AMPK by using an antibody against phosphorylated AMPK α at Thr172. b. AICAR increases mitochondrial mass in ATM+, but not ATM− cells. Cells were treated with 250 µM AICAR for 2 days. They were then fixed and stained by 50 µM Mitotracker Green FM followed by FACS analysis. c–d. AICAR induces ATM-dependent increase in mitochondrial membrane potential. Cells were treated with 250 µM AICAR for 2 days, and then stained with JC-1, followed by FACS analysis.

The AMPK activator AICAR can be converted to ZMP in cells to mimic AMP, and therefore creates a situation that mimics ATP reduction or high AMP/ATP ratio in cells [37], [38]. As shown in Fig 5a, AICAR increased phosphorylation of AMPK α subunit at Thr172 after 100 min in L40 (ATM+) cells, but not in L3 (ATM−) cells, consistent with a recent report that phosphorylation of the AMPK α subunit at Thr172 by AICAR requires ATM in MEF cells [26]. We further tested whether AICAR-induced mitochondrial biogenesis was affected by ATM. As shown in Fig. 5b, AICAR (250 µM) promoted mitochondrial mass increase in ATM+ L40 cells, but not in ATM- L3 cells. The increases in H population and the mean FL2 intensity of G1 population (healthy cells) induced by AICAR were also dependent on ATM (Fig. 5c and 5d). These results suggest that ATM is required for AICAR-induced AMPK activation and subsequent mitochondrial biogenesis. These results together with the observation that ATM phosphorylates AMPK [25], suggest that ATM could act as an upstream kinase of AMPK in the mitochondrial biogenesis pathway. It is possible that normal ATM activation at the end of G2 phase [39] or by hormone (serum) stimulations [25] may be responsible for the AICAR-induced AMPK/mitochondrial biogenesis. Clearly, further studies are needed to determine the mechanism of the ATM/AMPK/mitochondrial biogenesis pathway.

**Figure 6:**
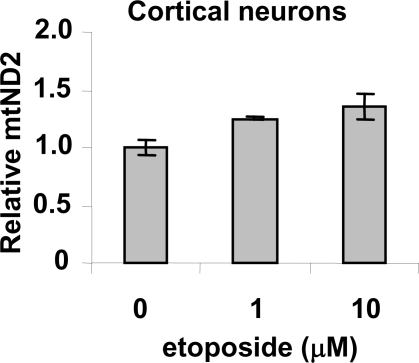
Etoposide increases mitochondrial DNA content in mouse cortical neurons. Isolated embryonic mouse cortical neurons (embryonic day 17.5) were treated with etoposide for 18 hrs. The relative amount of the mitochondrial mtND2 gene in mouse cortical neurons was measured by quantitative real-time PCR.

To test whether DNA damage-induced mitochondrial biogenesis is a general phenomenon in all cell types, post-mitotic cortical neurons were isolated from mouse embryos (embryonic day 17.5). As shown in Fig. 6, etoposide treatment induced an increase in mitochondrial DNA content in mouse cortical neurons. Since defects in activation of mitochondrial biogenesis and regulation of mitochondrial dynamics have been demonstrated to play important roles in neuronal degeneration [40]–[42], it is possible that the lack of mitochondrial biogenesis in response to DNA damage also contributes to the neuronal degeneration in A-T patients.

**Figure 7:**
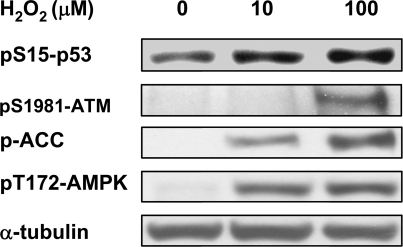
H_2_O_2_ treatment increases ATM autophosphorylation, and phosphorylations of p53, AMPK α and ACC (Acetyl-CoA Carboxylase). HeLa cells were treated with indicated concentrations of H_2_O_2_ for 16 hrs followed by cell lysis. Western blottings were performed using antibodies against phosphorylated ATM (Ser1981), p53 (Ser15), AMPK α (Thr172) and ACC (Ser79), respectively.

It has been shown that H_2_O_2_ can activate mitochondrial biogenesis [43]. To test whether this might also be through the ATM/APMK pathway, we monitored activation of ATM and AMPK. As shown in Fig. 7, H_2_O_2_ treatment activated ATM as evidenced by ATM autophosphorylation at Ser1981 and Ser15 phosphorylation of p53 (a substrate of ATM). H_2_O_2_ treatment also activated AMPK as evidenced by Thr172 phosphorylation of AMPK α and Ser79 phosphorylation of ACC (Acetyl-CoA Carboxylase, a substrate of activated AMPK). These results suggest that ROS might promote mitochondrial biogenesis through the DNA damage/ATM/AMPK pathway.

## Discussions

Previous studies have demonstrated that DNA damaging agents such as etoposide and doxorubicin induce mitochondrial biogenesis [21], [22]. In the current study, we show that etoposide-induced mitochondrial biogenesis is ATM-dependent as evidenced in three pairs of ATM-deficient cells. We have also provided evidence that ATM-dependent mitochondrial biogenesis is mediated through AMPK, an energy sensor known to be exquisitely sensitive to AMP/ATP ratio [19]. It has been reported that etoposide induces a drop in ATP pool [44]. This is further supported by the observation in bacteria that DNA damage induces a RecA-dependent drop in the ATP pool [45], [46]. It has also been demonstrated that ATM positively regulates the ATP pool since the ATP pool is significantly lower in A-T cells compared to ATM+ cells [47]. On the other hand, it has been demonstrated that ATM phosphorylates AMPK *in vitro* and activates AMPK through Thr172 phosphorylation *in vivo* [25], [26]. We have also shown that AICAR, which generates an ATP-deficient condition in cells, also requires ATM for mitochondrial biogenesis. It is likely that DNA damage induced activation of AMPK and subsequent mitochondrial biogenesis may be mediated through both the AMP/ATP ratio change and ATM activity (a proposed AMPK upstream kinase).

Mutation in ATM leads to the human disease *ataxia-telangiectasia* (A-T). The major clinical manifestation of A-T is ataxia, which is due to progressive cortical cerebellar degeneration primarily in Purkinje and granular cells [48]–[51]. In addition, A-T patients are immunodeficient and predisposed to cancers, particularly lymphomas and leukemia [52]–[58]. Furthermore, individuals with A–T exhibit premature aging, including elevated diabetes mellitus incidence and progeric hair and skin changes [48], [59]–[62]. This disease also manifests sensitivity to ionizing radiation [63]–[65].

ATM is known to be a key sensor for DNA double-strand breaks (DSBs) and is activated through autophosphorylation [23], [24], [66]. Upon activation, ATM phosphorylates a number of substrates for DNA repair, cell cycle regulation and apoptosis [67]–[69]. Growing evidence has suggested that ATM plays an important role in oxidative stress [70]. Individuals with A–T exhibit elevated oxidative stress as compared to normal controls, including increased oxidative damage to DNA and lipids, and antioxidant defense alterations secondary to oxidative stress [71], [72]. ATM-deficient mice similarly exhibit elevated oxidative stress [73]–[77]. Interestingly, administration of antioxidant (e.g. EUK-189 and NAC) to ATM-deficient mice lowers oxidative DNA, lipid, and protein damages, reduces cancer incidences, inhibits cerebellar Purkinje cell death, and partially reverses the neurological deficits and premature aging seen in these mice [73], [78]–[81]. However, the molecular basis for increased ROS in A-T cells is still unclear.

ROS is known to be primarily produced in mitochondria due to incomplete reduction of oxygen during oxidative phosphorylation for ATP production. Defects in mitochondrial function such as inefficient oxidative phosphorylation and loss of membrane potential can lead to elevated ROS production. Mitochondrial quality is maintained through biogenesis of functional mitochondria, as well as turnover of defective mitochondria [82]. We have shown that ROS (H_2_O_2_) can activate ATM and AMPK (Fig. 7). It has also been reported that H_2_O_2_ increases mitochondrial biogenesis [43]. These results suggest that ROS might increase mitochondrial biogenesis through a DNA damage/ATM/AMPK pathway. We therefore propose that ROS may perturb mitochondrial homeostasis through two opposing effects. First, ROS can damage mitochondria leading to the production of more ROS and more defective mitochondria via a vicious cycle. Second, ROS can also induce mitochondrial biogenesis through a DNA damage/ATM/AMPK pathway and therefore ameliorate the ROS-mediated vicious cycle. In A-T cells, the lack of ATM/AMPK/mitochondrial biogenesis leads to elevated production of ROS due to the unchecked vicious cycle. The elevated ROS will result in more DNA damage (in both mitochondrion and nucleus), and eventually depolarization of mitochondria (as seen in Fig. 3a) and cell death. The fact that ATM deficient cells are also defective in DNA damage repair could worsen the situation.

Based on this view, mitochondrial biogenesis may be critical for controlling ROS production and ROS-mediated DNA damage. It is possible that defect in ATM/AMPK/mitochondrial biogenesis in A-T patients may contribute significantly to some of the A-T manifestations which can be reversed by antioxidants. Cerebellar Purkinje cell death and neurological deficits in A-T mice have been shown to be inhibited by antioxidants [60], [78]. We have shown (Fig. 6) that DNA damage induced by etoposide treatment stimulates mitochondrial biogenesis in mouse neuronal cells. It is tempting to speculate that lack of the ATM/AMPK/mitochondrial biogenesis pathway may be a major cause for the progressive cerebellar degeneration in A-T patients. Interestingly, defects in mitochondria and mitochondrial biogenesis have been linked to neurodegenerative diseases, diabetes mellitus and other aging diseases [1]–[4], [40]–[42]. Furthermore, antioxidants have also been shown to suppress cancer incidences, inhibit immunodeficiency and extend lifespan in A-T mice, in addition to neurological deficits [73], [78]–[81]. It is possible that defect in mitochondrial biogenesis plays an important role in ROS regulation and the development of A-T disease. Thus, mitochondrial biogenesis can be targeted for its therapy.

It has been reported that ATM can also be activated by certain dietary components (e.g. resveratrol in red wine, benzyl isothiocyanate (BITC) in papaya and certain vegetables and the micronutrient selenium) [83]–[85] that are known to have anti-aging or anticancer activities. It is tempting to speculate that these dietary components may stimulate mitochondrial biogenesis through ATM to maintain functional mitochondria and regulate ROS. Clearly, more studies are needed to establish the role of ATM-dependent mitochondrial biogenesis in dietary modulation of cancer and aging.

## Materials and Methods

### Reagents

Etoposide (VP16) was purchased from Sigma-Aldrich (St. Louis, MO). The fluorescent dyes 10-n-Nonyl-Acridine Orange (NAO), MitoTracker Green FM, and 5,5′,6,6′-tetrachloro-1,1′,3,3′-tetraethylbenzimidazole carbocyanide iodide (JC-1) were purchased from Invitrogen (Carlsbad, CA). Caspase inhibitor z-VAD-FMK was purchased from Promega (Madison WI). The primer and probe set for detecting human mitochondrial NADH dehydrogenase subunit 2 (mtND2) and the endogenous control for detecting 18S rDNA were purchased from Applied Biosystems (Foster City, CA). Antibodies against AMPK α and phosphorylated ATM at Ser1981, p53 at Ser15, AMPK at Thr172 and ACC at Ser79 were purchased from Cell Signaling (Danvers, MA). Monoclonal antibody against α-tubulin was obtained from Sigma-Aldrich. Compound C was a kind gift from Dr. Gaoqiao Zhou (Merck Research Laboratories, Rahway, NJ). siRNA against AMPK α1 subunit (sc-29673) was purchased from Santa Cruz Biotechnology, Inc (Santa Cruz, CA).

### Cell Culture

HeLa cells were purchased from American Type Culture Collection (Manassas, VA), and were cultured in complete DMEM (DMEM supplemented with 10% FCS, 100 units/ml penicillin, 100 µg/ml streptomycin and 2 mM glutamine). The transformed FT169A A-T fibroblasts stably transfected with plasmid vector pEB7 (ATM−) and the ATM cDNA YZ5 (ATM+) (obtained from Dr. Y. Shiloh, Tel Aviv University, Israel) [86] were cultured in complete DMEM supplemented with 100 µg/ml hygromycin. The HeLa ATM601 (ATM siRNA) and its control NS siRNA (Non Specific siRNA) cell lines (obtained from Dr. B.D. Price, Harvard Medical School, Boston, MA) [87] were cultured in complete DMEM supplemented with 400 µg/ml G418. Lymphoblastoid L40 (ATM+) and L3 (ATM−) (obtained from Dr. Yosef Shiloh) [88] were cultured in RPMI medium supplemented with 10% FCS, 100 units/ml penicillin, 100 µg/ml streptomycin and 2 mM glutamine. Mouse cortical neurons were cultured in neurobasal medium (NBM, Invitrogen, Carlsbad, CA) supplemented with B27 (Invitrogen, Carlsbad, CA), 0.5 mM Glutamine, 100 units/ml penicillin and 100 µg/ml streptomycin. All cells were cultured in a humidified atmosphere consisting of 95% air and 5% CO_2_.

### Measurement of Mitochondrial DNA Content

Cells were seeded at a density of 50,000 cells per dish in 35 mm dishes one day prior to drug treatment. Etoposide treatment usually lasts for 16 hrs unless indicated otherwise. After the treatment, cells were washed twice with Phosphate Buffered Saline (PBS, pH 7.2) and lysed in 100 µl lysis buffer (1 mM EDTA, 10 mM Tris, pH 8.0, 1% SDS). Cell lysates were then denatured at 95°C for 20 min and diluted 25-folds with water. Four microliters of the diluted cell lysates were used for the quantitative real time PCR reaction. Mitochondrial DNA was quantified using a probe that specifically recognizes the mitochondrial gene MtND2. The mouse mtND2 primer set and probe were designed using Primer Express software and synthesized by Applied Biosystems (Foster City, CA). The detection sequences are as follows:

1. Forward primer: 5′-AACCCACGATCAACTGAAGCA-3′
2. Reverse primer: 5′-ACGATGGCCAGGAGGATAATT-3′
3. TaqMan probe: 5′-AATACTTCGTCACACAAGCA-3′

The amount of 18S rDNA in the same reaction was simultaneously determined and used as the internal control. The quantitative real time PCR reaction was carried out in a total volume of 20 µl using TaqMan reagents following the manufacturer's instructions (Applied Biosystem, TaqMan Universal PCR Master Mix). Sequence-specific amplification was detected on the ABI Prism 7900 sequence detection system (Applied Biosystems). Data were extracted and analyzed using the SDS2.1 software (Applied Biosystem).

### Determination of Mitochondrial Mass

#### NAO-staining

The fluorescent dye 10-N-Nonyl-Acridine Orange (NAO) binds specifically to cardiolipin at the inner mitochondrial membrane independently of membrane potential and was used to monitor the mitochondrial mass as described before [22]. Once treatment was completed, cells were trypsinized and stained with 1 µM NAO at room temperature in the dark for 10 min, followed by FACS analysis. The mean fluorescence intensity was plotted against each treatment. Mitochondrial mass was also measured by **Mitotracker Green FM staining.** Treated cells were fixed by 60% ethanol at 4°C. After washing with PBS, fixed cells were stained by 50 µM Mitotracker Green FM followed by FACS analysis.

### RT-PCR

Total RNA was isolated using Trizol reagent (Invitrogen) and cleaned with the RNeasy Mini Kit (Qiagen) according to the manufacturer's instructions. About 1–2 µg RNA was used for the synthesis of first strand cDNA using the SuperScript III First-Strand Synthesis System (Invitrogen). PCR reaction was performed on a PTC-100 Thermal Cycler (MJ Research) and PCR products were analyzed by electrophoresis on a 2% agarose gel. The images of ethidium bromide-stained agarose gels were captured on the Kodak Image Station 2000R. The band intensity of PCR products were quantified using the KODAK 1D 3.6 software.

The specific primers used are as follows: 5′-TGGGACAGCAAGCTATTGTCCTCT-3′, NRF-1 forward primer. 5′-ACTGGAATTCCGTCGATGGTGAGA-3′, NRF-1 reverse primer. 5′-ACCTGACACAACACGGACAGAACT-3′, PGC-1α forward primer. 5′-TCTTGGTGGAAGCAGGGTCAAAGT-3′, PGC-1α reverse primer. 5′-ACAGCTAACTCCAAGTCAGATTATGTC-3′, TFAM forward primer. 5′-GTAGACACTTGAGACTAACAACCGT-3′, TFAM reverse primer. 5′CAAAGACCTGTACGCCAACACAGT3′, β-Actin forward primer. 5′-TTGCTGATCCACATCTGCTGGAAG-3′, β-Actin reverse primer.

### Determination of Mitochondrial Membrane Potential

JC-1 was added to treated cells to a final concentration of 2.5 µg/ml. Cells were stained in the dark at 37°C for 15 min. They were then washed with PBS once, followed by FACS analysis. Photomultiplier settings were adjusted to detect green fluorescence (λ_em_ = 525 nm) of JC-1 monomer using filter 1 (FL1 detector) and the red fluorescence (λ_em_ = 590 nm) of JC-1 aggregates using filter 2 (FL2 detector). In each experiment, at least 10,000 events were analyzed. Data were collected from normal cell population for each sample, which was gated according to the no treatment controls based on forward and side scatters. The ratios of JC-1 aggregate/monomer (red/green or FL2/FL1) reflect mitochondrial membrane potential.

### Isolation of Mouse Cortical Neurons

All mice were maintained in accordance with the guidelines of the Institutional Animal Care and Use Committee (IACUC) at University of Medicine and Dentistry at New Jersey (UMDNJ)-Robert Wood Johnson Medical School. Mouse cortical neurons were isolated from E17.5 C57/BL6 mouse embryos (embryonic day 17.5) as described [89]. Briefly, cerebral cortex was removed from mouse embryos and the surrounding meninges/blood vessels were peeled off to minimize contamination from endothelial cells. The brain tissue was minced and digested with trypsin. The trypsinization reaction was stopped with soybean trypsin inhibitor and the mixtures were digested with DNase I. The remaining tissue was triturated gently through a fire-polished Pasteur pipette and cells were collected from the resulting homogeneous suspension by centrifugation. The isolated mouse cortical neurons were then plated on Poly-L-lysine (PLL)-coated tissue culture plates and allowed to recover for 48 hrs prior to various treatments.

### MTT assay

About 3000 cells per well (YZ5 or pEBS7) were seeded in 96-well plates and cultured overnight for re-attachment. Cells were then treated with indicated concentrations of etoposide for 18 hrs followed by 3-day culture in fresh medium. MTT assay was performed as described in [90]. In brief, cells were stained with 0.1 mg/ml MTT (Sigma) for 4 hrs and then dissolved in DMSO. MTT values were measured at 570 nm by a Biorad 3550 microplate reader.

## Supporting Information

Figure S1 Activation of AMPK by etoposide is ATM-dependent. YZ5 cells (A-T cells stably transfected with the ATM cDNA expression plasmid) and pEBS7 cells (A-T cells stably transfected with the vector) were treated with indicated concentrations of etoposide for 16 hrs and activation of AMPK was monitored using an antibody specific to phosphorylated AMPK a at Thr-172.
(4.01 MB TIF)

Figure S2 Etoposide-induced increase in mitochondrial DNA content is ATM-dependent. ATM- cells (AT cells stably transfected with the vector, pEBS7) and ATM+ cells (AT cells stably transfected with the ATM cDNA expression plasmid, YZ5) were treated with indicated concentrations of etoposide and the amounts of the mitochondrial mtND2 gene were determined by quantitative real time PCR.
(1.76 MB TIF)

Figure S3 Etoposide-induced increase in mitochondrial mass is ATM-dependent. L3 (ATM−) and L40 (ATM+) lymphoblastoid cells were treated with etoposide for 2 days and fixed with 60% ethanol. Mitochondrial mass was determined by MitoTracker Green FM staining and FACS analysis.
(5.04 MB DOC)

Figure S4 ATM improves mitochondrial membrane potential in surviving cells. L3 (ATM−) and L40 (ATM+) cells were treated with indicated concentrations of etoposide for two days, followed by JC-1 staining and FACS analysis.
(10.31 MB TIF)

Figure S5 ATM+ cells are more resistant to etoposide than ATM− cells. L40 (ATM+) and L3 (ATM−) cells were treated with etoposide for 18 hrs. Cells were then replenished with fresh medium and incubated for additional 3 days before MTT assay was performed. For MTT assay, cells were stained with 0.1 mg/ml MTT (Sigma) for 4 hrs and then dissolved in DMSO. MTT values were measured at 570 nm by a Biorad 3550 microplate reader.
(2.42 MB TIF)
