# Supplementary figures and images for: Etoposide Induces ATM-Dependent Mitochondrial Biogenesis through AMPK Activation
Source: PLoS One. 2008 Apr 23;3(4):e2009. doi: 10.1371/journal.pone.0002009 (PMC2329593; doi:10.1371/journal.pone.0002009)

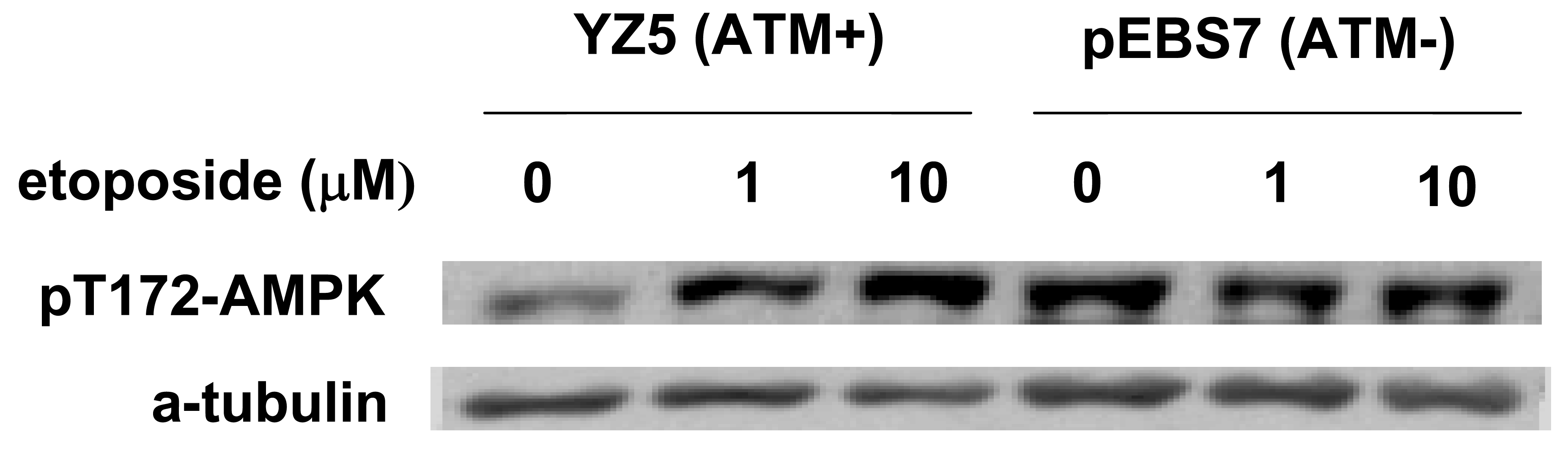

Supplement: Figure S1 — Activation of AMPK by etoposide is ATM-dependent. YZ5 cells (A-T cells stably transfected with the ATM cDNA expression plasmid) and pEBS7 cells (A-T cells stably transfected with the vector) were treated with indicated concentrations of etoposide for 16 hrs and activation of AMPK was monitored using an antibody specific to phosphorylated AMPK a at Thr-172. (4.01 MB TIF) [file pone.0002009.s001.tif]

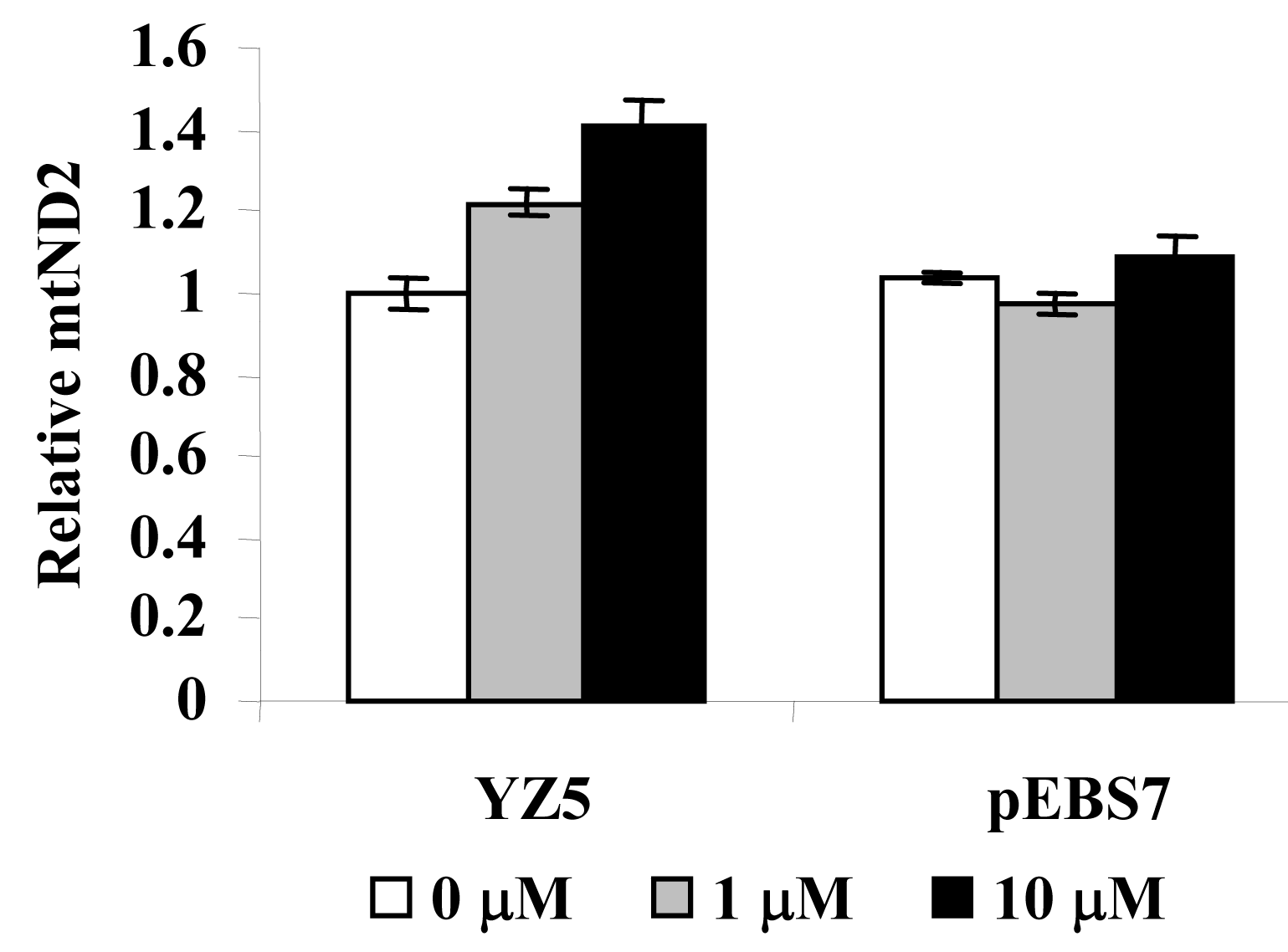

Supplement: Figure S2 — Etoposide-induced increase in mitochondrial DNA content is ATM-dependent. ATM- cells (AT cells stably transfected with the vector, pEBS7) and ATM+ cells (AT cells stably transfected with the ATM cDNA expression plasmid, YZ5) were treated with indicated concentrations of etoposide and the amounts of the mitochondrial mtND2 gene were determined by quantitative real time PCR. (1.76 MB TIF) [file pone.0002009.s002.tif]

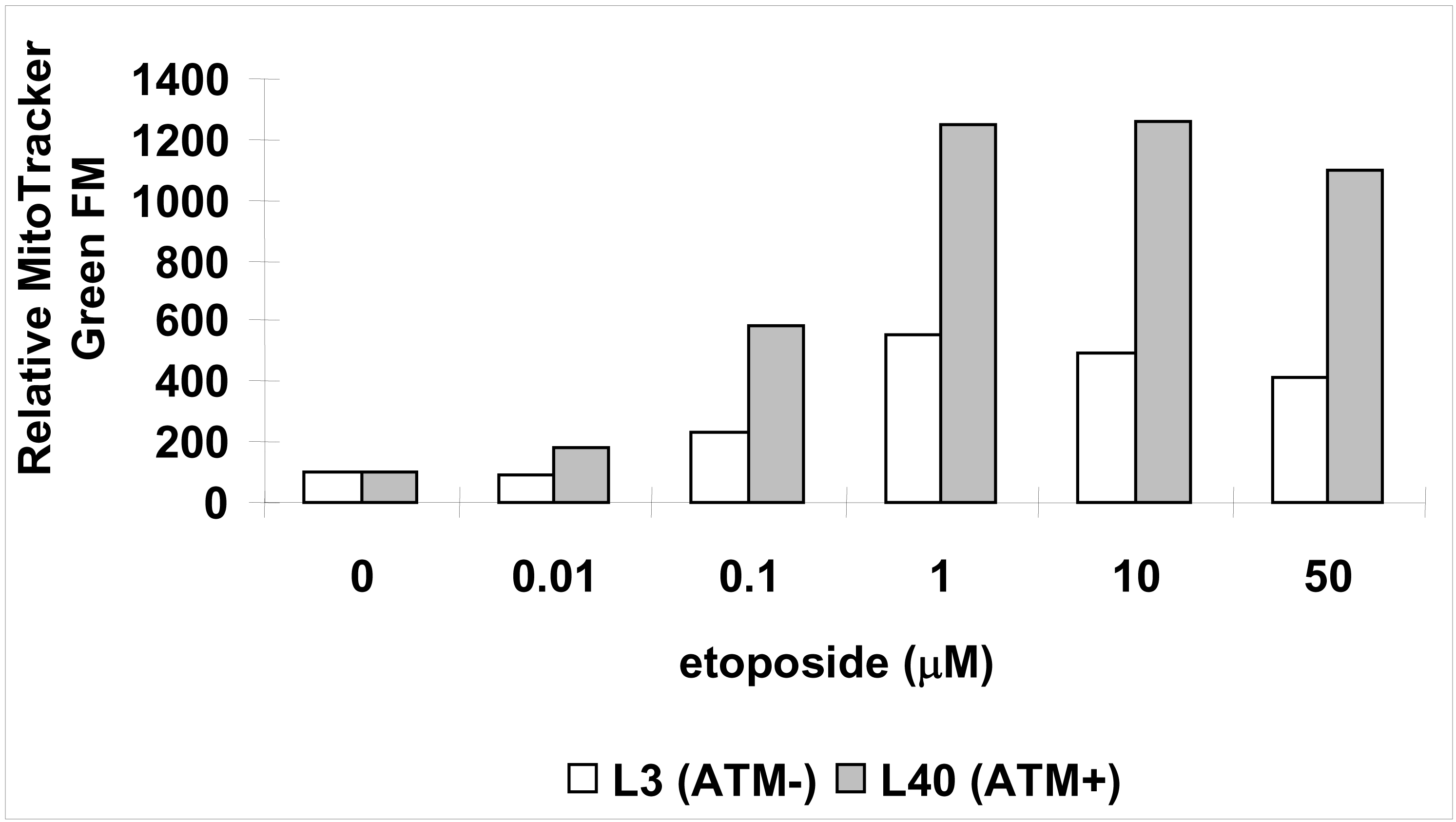

Supplement: Figure S3 — Etoposide-induced increase in mitochondrial mass is ATM-dependent. L3 (ATM−) and L40 (ATM+) lymphoblastoid cells were treated with etoposide for 2 days and fixed with 60% ethanol. Mitochondrial mass was determined by MitoTracker Green FM staining and FACS analysis. (5.04 MB DOC) [file pone.0002009.s003.tif]

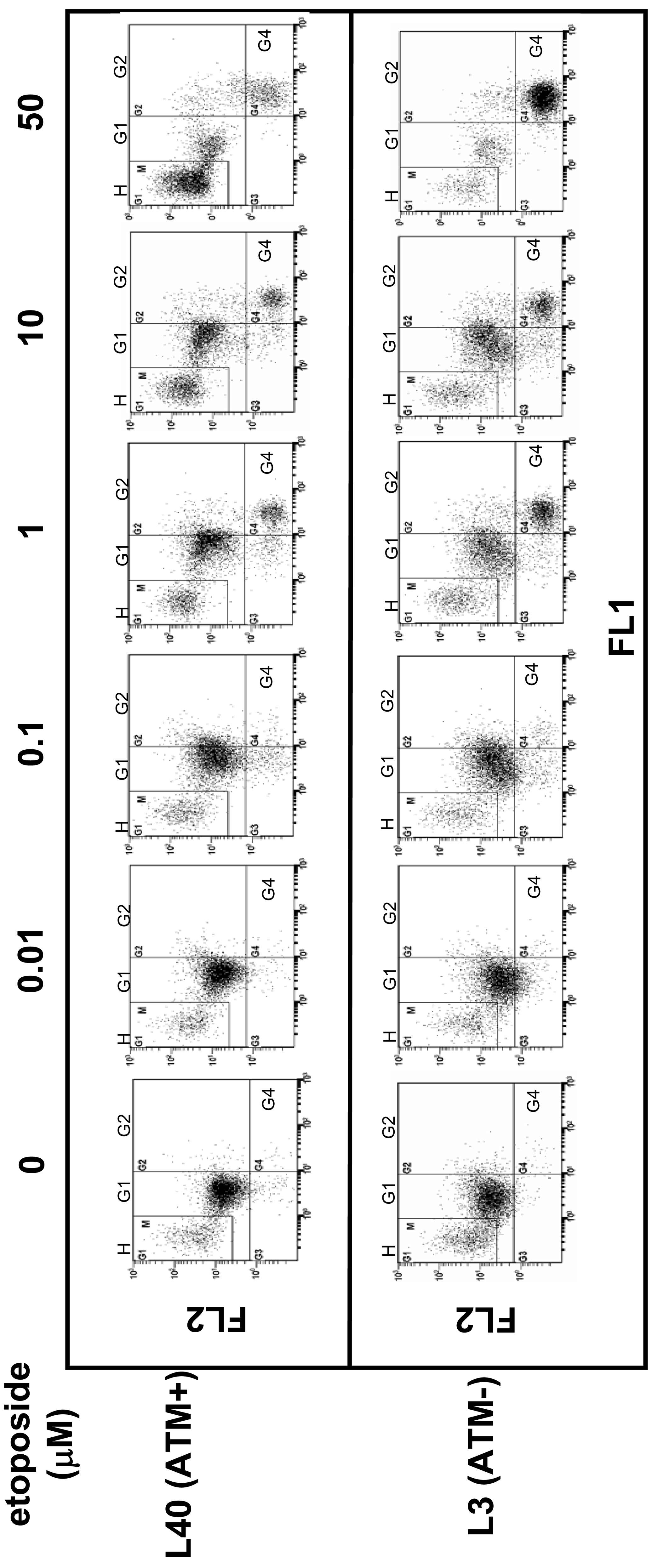

Supplement: Figure S4 — ATM improves mitochondrial membrane potential in surviving cells. L3 (ATM−) and L40 (ATM+) cells were treated with indicated concentrations of etoposide for two days, followed by JC-1 staining and FACS analysis. (10.31 MB TIF) [file pone.0002009.s004.tif]

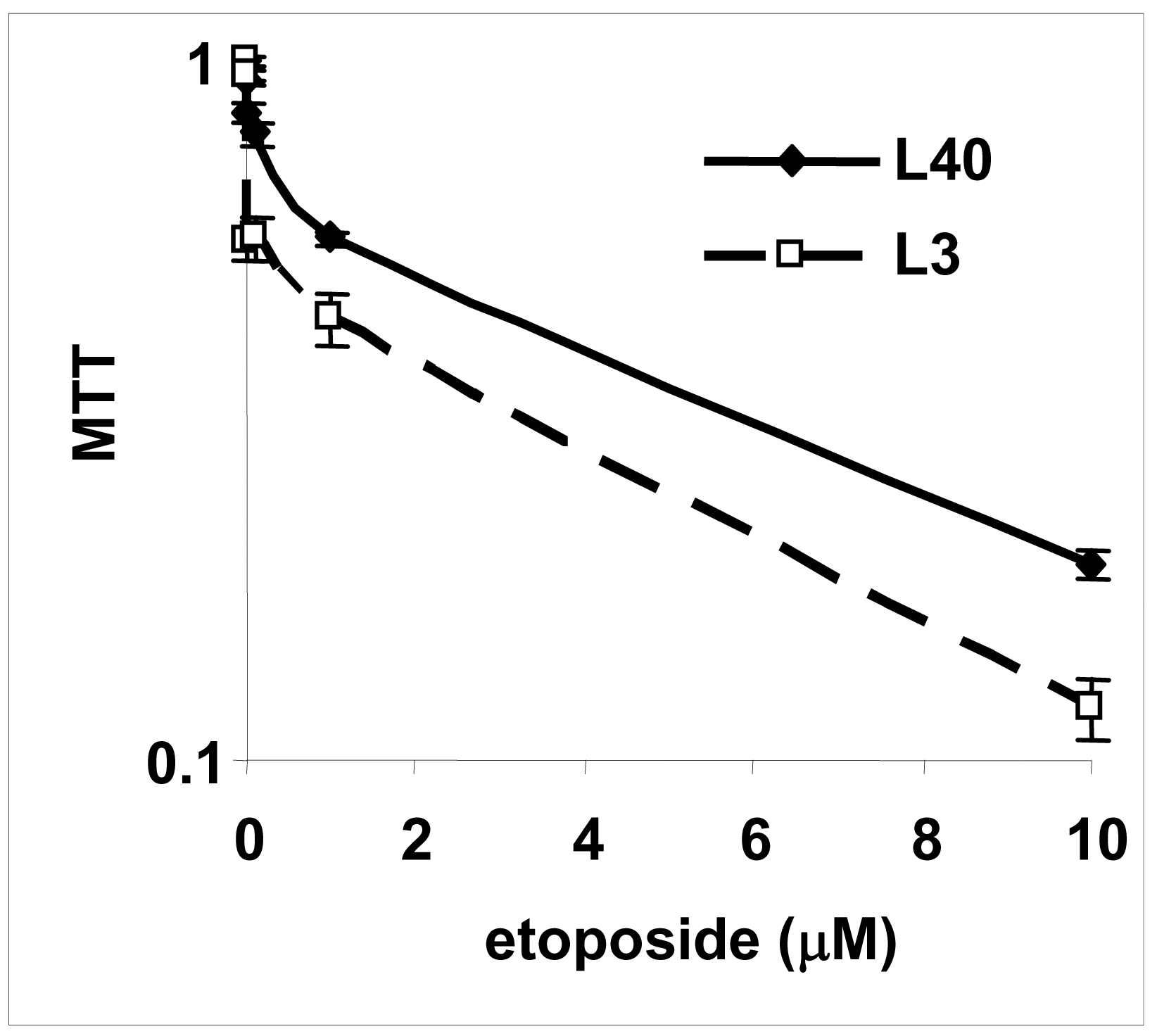

Supplement: Figure S5 — ATM+ cells are more resistant to etoposide than ATM− cells. L40 (ATM+) and L3 (ATM−) cells were treated with etoposide for 18 hrs. Cells were then replenished with fresh medium and incubated for additional 3 days before MTT assay was performed. For MTT assay, cells were stained with 0.1 mg/ml MTT (Sigma) for 4 hrs and then dissolved in DMSO. MTT values were measured at 570 nm by a Biorad 3550 microplate reader. (2.42 MB TIF) [file pone.0002009.s005.tif]
